# Supplementary material for: Improved calcium sensor GCaMP-X overcomes the calcium channel perturbations induced by the calmodulin in GCaMP
Source: Nat Commun. 2018 Apr 17;9:1504. doi: 10.1038/s41467-018-03719-6 (PMC5904127; doi:10.1038/s41467-018-03719-6)
Supplement: Supplementary file 1 — Supplementary Information [file 41467_2018_3719_MOESM1_ESM.pdf]

Supplementary Information

Improved calcium sensor GCaMP-X overcomes the calcium channel perturbations induced by the calmodulin in GCaMP

Yang *et al.*

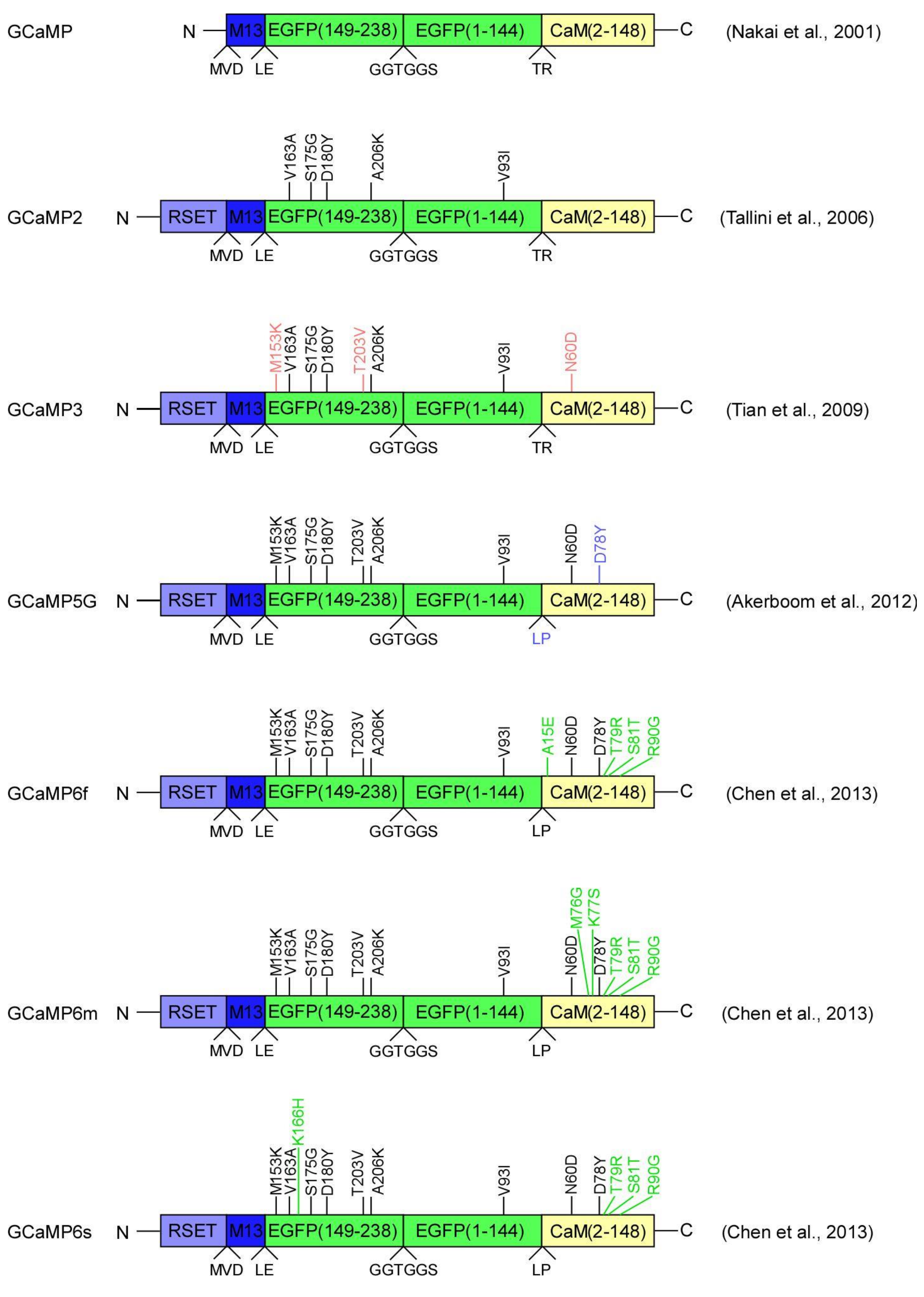

Supplementary Fig. 1 Sequence comparison among GCaMP series.

Updates of GCaMP series (with references for each version, right) were mostly achieved by mutations of EGFP, CaM and the linker in between. Mutations are indicated by vertical letters. Colored letters are to highlight new mutations of the later version of GCaMP compared to its previous version.

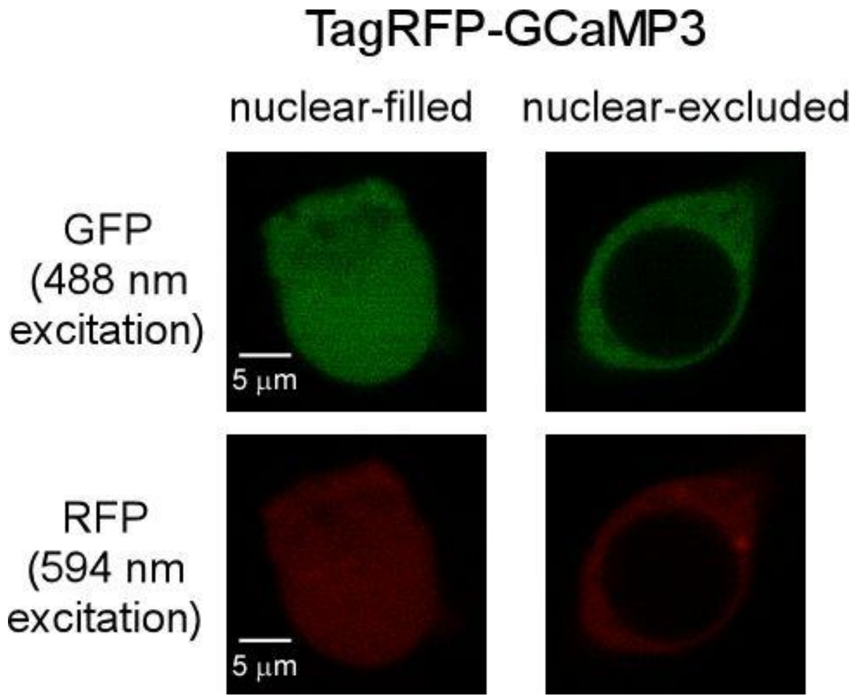

Supplementary Fig. 2 GCaMP3 distributions confirmed with TagRFP-GCaMP3.

GCaMP3 was fused with a fluorescent protein TagRFP, which emits red fluorescence independent of Ca^{2+}. Cortical neurons expressing TagRFP-GCaMP3 were observed with 488 nm laser (excitation wavelength of GCaMP3) and 594 nm laser (excitation wavelength of TagRFP). Fluorescence of GCaMP3 was colocalized with TagRFP, similarly exhibiting both nuclear-filled and nuclear-excluded patterns.

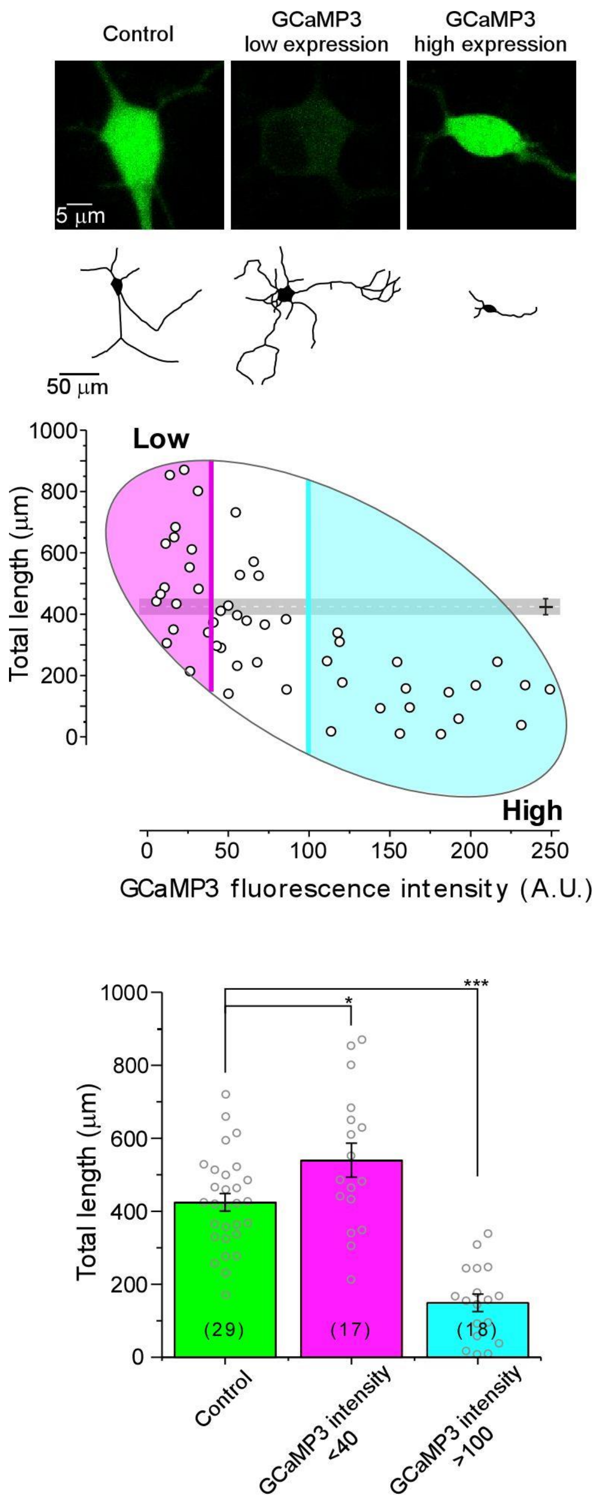

Supplementary Fig. 3 Correlation between GCaMP expression level and neurite length.

Similar to the criteria and analyses with the index of N/C ratio (**Fig. 1g**), cultured cortical neurons (DIV 5) transfected with GCaMP3 are categorized into two major subgroups: low expression group (GCaMP3 fluorescence intensity < 40, in arbitrary units, A.U.) and high expression group (intensity > 100). Representative images of fluorescence intensity (green) and tracing neurite morphology (black and white) are shown (top). GCaMP3 fluorescence intensity and total neurite length for each individual neuron are examined for their potential correlation (middle). Individual neurons with low expression (pink area) or

high expression (cyan area) levels of GCaMP3 are grouped respectively according to the above criteria. Grey line/area represents the total neurite length per neuron for the control group. The eclipse enclosing all of the data points illustrates a strong (negative) correlation between GCaMP3 fluorescence intensity and total neurite length per neuron (correlation coefficient= –0.7). Analyses on total neurite length per neuron for the three groups (bottom) confirm that the average length of low-expression group is significantly less than that of control group, whereas the high-expression group has significantly larger value of average neurite length than the control group.

Standard error of the mean (S.E.M.) and Student's *t*-test (two-tailed unpaired with criteria of significance: *, $p<0.05$; **, $p<0.01$ and ***, $p<0.001$) were calculated when applicable.

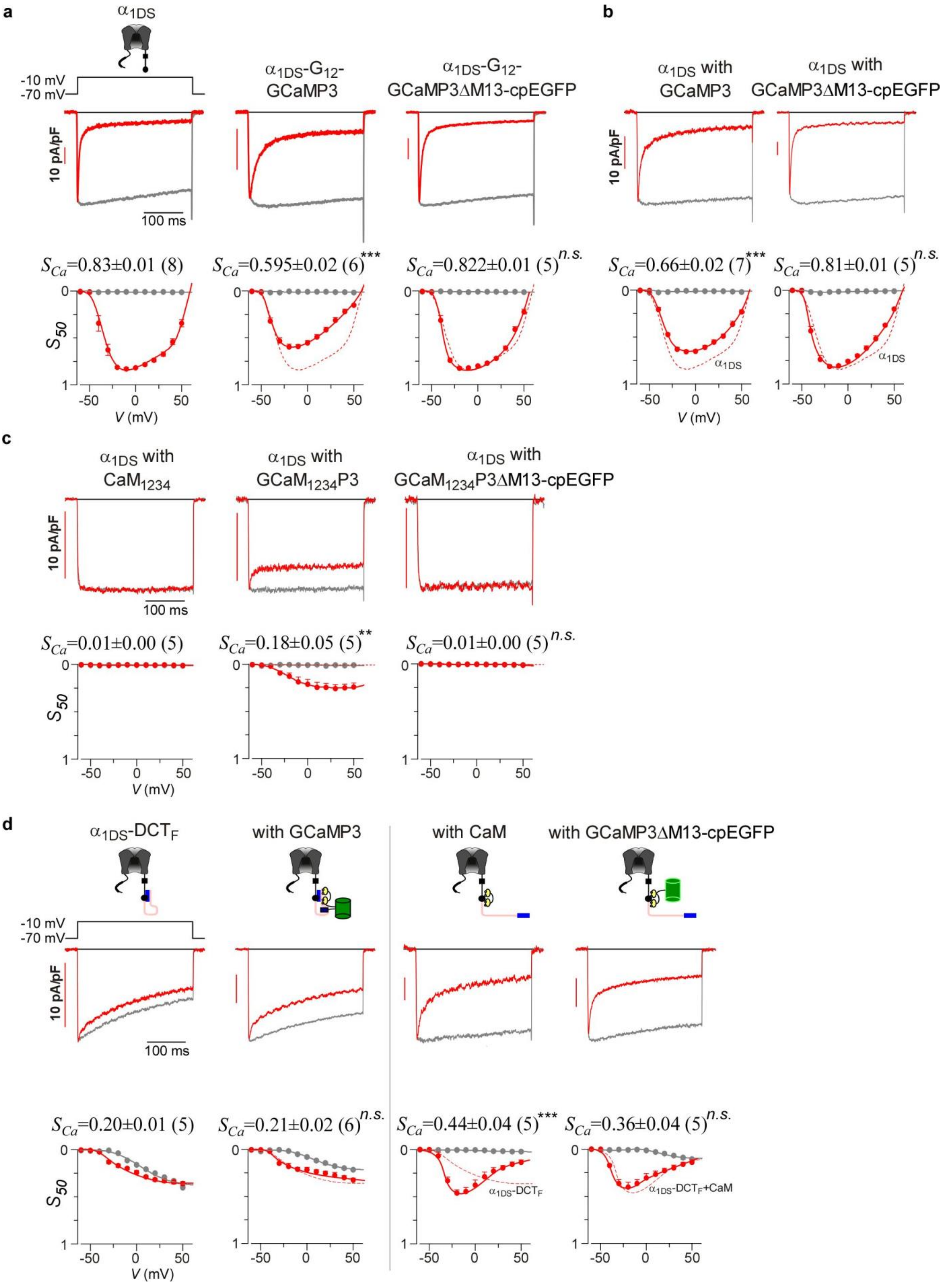

Supplementary Fig. 4 Details on CaM properties contained within GCaMP.

(**a**) Chimeric channels revealed that M13 and cpEGFP in GCaMP3 lead to aberrant function of CaM in GCaMP3. Representative recording exemplars in HEK293 cells expressing short variant of $Ca_V1.3$ channels alone (α_{1DS}) (left), α_{1DS}-G_{12}-GCaMP3 (middle) or α_{1DS}-G_{12}-GCaMP3ΔM13-cpEGFP (right), respectively. Protocols, indices and styles are all similar to Fig. 2c, d. Briefly, inactivation profiles across the full voltage range (V) for I_{Ba} and I_{Ca} were quantified by S_{50} ($S_{50} = 1 - I_{50}/I_{peak}$). The CDI strength ($S_{Ca}$) were quantified by S_{50} in Ca^{2+} at −10 mV, as the index for each CDI profile. Statistical significance (unpaired Student's t-test) was evaluated by comparing each experimental group with the control (dotted line). (**b**) Further consolidation by co-expression of GCaMP3 or GCaMP3ΔM13-cpEGFP with α_{1DS}. Representative recording traces and CDI profiles were shown with identical manner in (**a**). (**c**) Representative recording exemplars in HEK293 cells expressing short variant of $Ca_V1.3$ channels with CaM_{1234} (left), $GCaM_{1234}P3$ (middle) and CaM_{1234} from $GCaM_{1234}P3$ (right), respectively. CaM_{1234} mutant loses function of calcium binding, leading to abolishment of CDI. CaM_{1234} and the CaM_{1234} from $GCaM_{1234}P3$ competed with endogenous CaM and abolished endogenous CaM-mediated CDI completely. Whereas $GCaM_{1234}P3$ still remained partial CDI. (**d**) Further exploration of GCaMP function using α_{1DS}-DCT_F. Distal carboxyl tail from $Ca_V1.4$ (α_{1F}) was fused to short variant of $Ca_V1.3$ (α_{1DS}) to construct chimeric channels α_{1DS}-DCT_F. With competition between DCT_F and apoCaM for IQ region of α_{1DS}, CDI from α_{1DS}-DCT_F were substantially attenuated. GCaMP3 could not function as a normal CaM to enhance CDI of α_{1DS}-DCT_F. But without M13 and cpEGFP, the CaM within GCaMP3 could enhance CDI of α_{1DS}-DCT_F as well as wild type CaM.

Standard error of the mean (S.E.M.) and Student's t-test (two-tailed unpaired with criteria of significance: *, $p<0.05$; **, $p<0.01$ and ***, $p<0.001$) were calculated when applicable, and *n.s.* denotes "not significant".

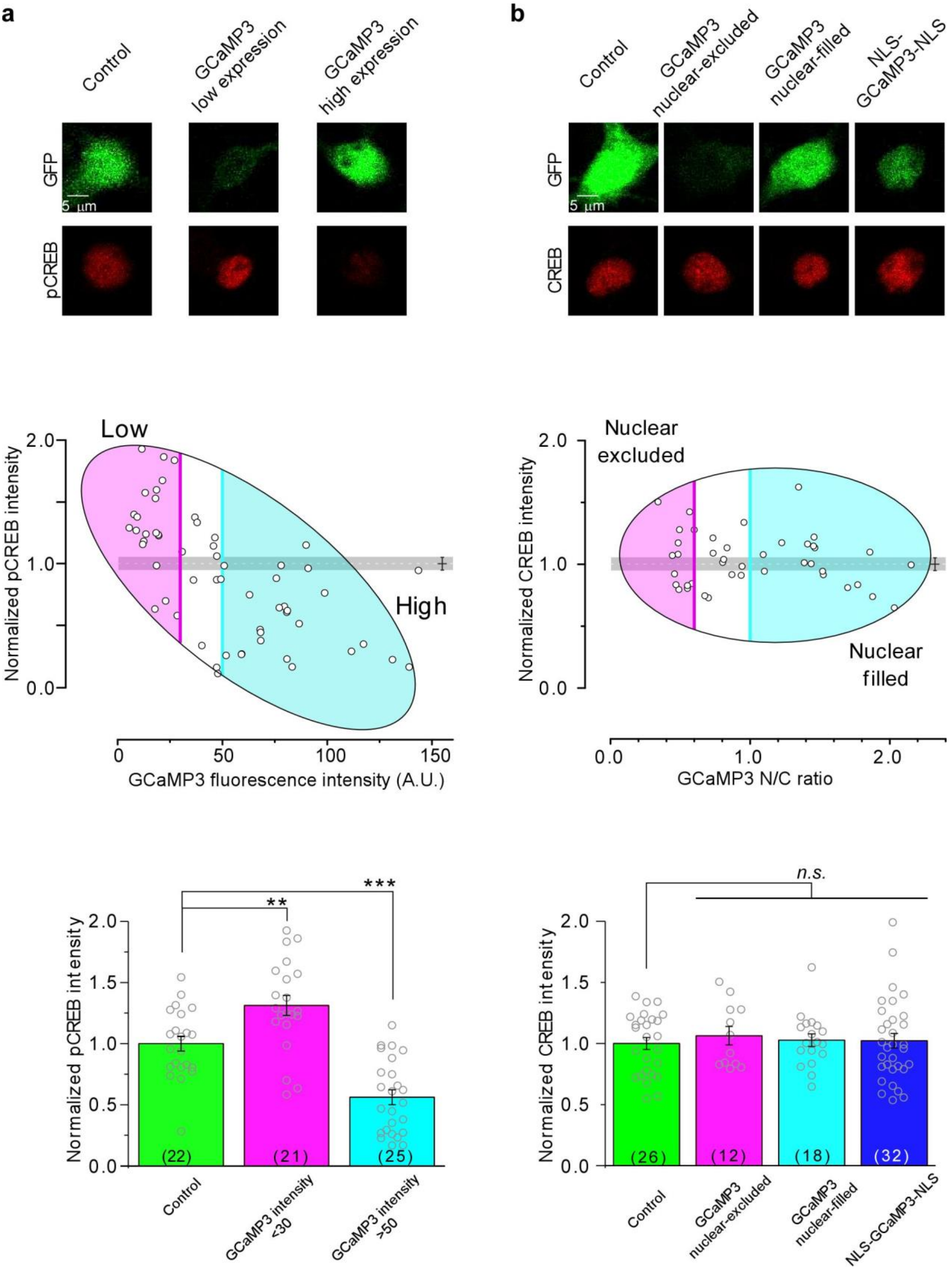

Supplementary Fig. 5 To correlate expression level or N/C ratio of GCaMP with pCREB or total CREB. (**a**) GCaMP levels highly correlate with pCREB signals. DIV 5 cortical neurons were transfected with GCaMP3 and fixed in DIV 7 for immunostaining with pCREB-specific antibodies (exemplar images shown on top). Compared with the control group (YFP only), GCaMP-expressing neurons were categorized into low expression and high expression groups, by the criteria of fluorescence intensity <30 and >50 in arbitrary units (A.U.) respectively, similar to Supplementary Fig. 3 but overall with lower

level of fluorescence intensity due to cell fixation. The tilted eclipse enclosing all individual neurons (middle) indicates a strong (negative) correlation between GCaMP3 fluorescence intensity and pCREB signal (correlation coefficient = −0.6). Neurons of low-expression and high-expression groups exhibited significantly up- or down-regulated pCREB signals (bottom). (**b**) No correlation between N/C ratio of GCaMP and total CREB. Similar experiments and analyses as in (**a**) were performed, except for a different kind of antibodies here to stain total CREB (instead of pCREB specifically). The correlation coefficient between GCaMP3 N/C ratio and CREB signal is rather low (~ 0.06) (middle). Among all the four groups: control (YFP only), nuclear-filled and nuclear-excluded (by N/C ratio), and constitutively nuclear-filled (NLS-GCaMP-NLS), no significant difference in CREB level was evidenced (bottom). Standard error of the mean (S.E.M.) and Student's *t*-test (two-tailed unpaired with criteria of significance: *, $p<0.05$; **, $p<0.01$ and ***, $p<0.001$) were calculated when applicable, and *n.s.* denotes "not significant".

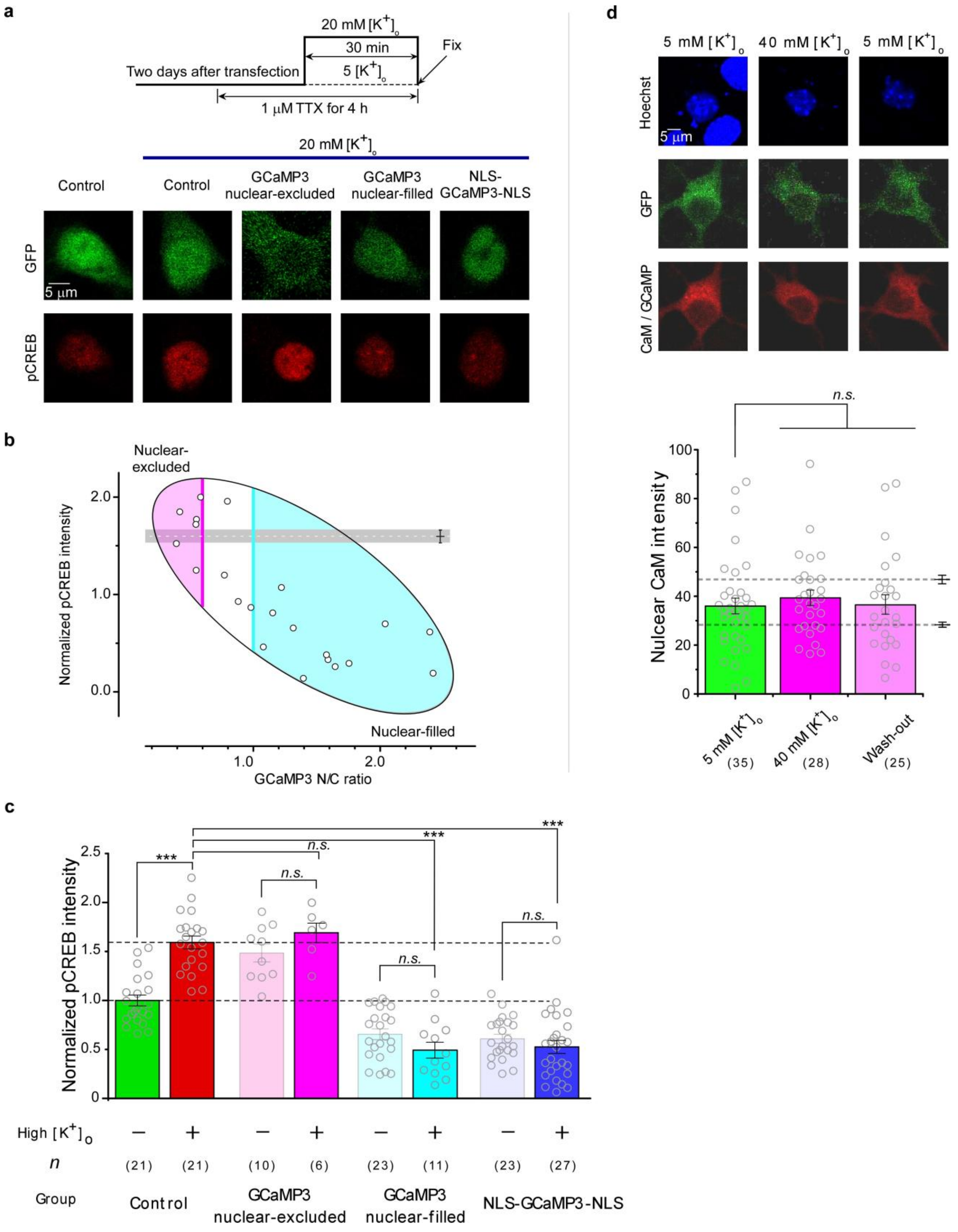

Supplementary Fig. 6 GCaMP3 perturbs transcription signaling in response to membrane excitation. (**a**) DIV 5 cortical neurons were transfected with YFP, GCaMP3 or NLS-GCaMP3-NLS for 2 days, and were silenced by 1 μM TTX for 4 hours before stimulation. Then neurons were stimulated with 20 mM $[K^+]_o$ (containing 1 μM TTX) for 30 min, and immediately fixed. Green fluorescence (upper) illustrates the distributions of overexpressing proteins and red staining (lower) represents the level of pCREB. (**b**) The correlation between N/C ratio of GCaMP3 and normalized pCREB intensity.

Cortical neurons with or without GCaMP3 were examined by 20 mM $[K^+]_o$ protocol as in (**a**). GCaMP3-expressing neurons were categorized into two subgroups of nuclear-excluded or nuclear-filled, indexed by GCaMP3 N/C ratio. (**c**) Statistical summary of pCREB intensity in cortical neurons. Control group neurons exhibited significant up-regulation of pCREB intensity under high potassium stimulation, whereas other three groups including NLS-GCaMP3-NLS, GCaMP3 nuclear-filled group and GCaMP3 nuclear-excluded group, did not respond to high $[K^+]_o$. All group were normalized to average pCREB intensity of control group (neurons with YFP). (**d**) Cytonuclear translocations of endogenous CaM was impaired in GCaMP3-expressing neurons. The experimental protocol was identical to Fig. 3d, but CaM staining here indicated both (nuclear) endogenous CaM and (cytosolic) GCaMP for the nuclear-excluded group of neurons. Nuclear CaM fluorescence intensity (in A.U.) instead of N/C ratio was used to depict endogenous CaM translocation. GCaMP would not translocate into the nucleus (Fig. 3e, f) and the nuclear fluorescence change should indicate the potential translocation of endogenous CaM. Nuclear CaM intensities for the three groups (resting, high $[K^+]_o$ stimulus and washout) did not exhibit any significant change, suggesting that the acute mobility of endogenous CaM was significantly impaired by GCaMP3 in neurons. The dotted lines represent the fluorescence intensity levels of nuclear CaM staining in control neurons, at rest (28.5 ± 1.1, n=69) and in 40 mM $[K^+]_o$ (46.7 ± 1.8, n=59). Standard error of the mean (S.E.M.) and Student's *t*-test (two-tailed unpaired with criteria of significance: *, $p<0.05$; **, $p<0.01$ and ***, $p<0.001$) were calculated when applicable, and *n.s.* denotes "not significant".

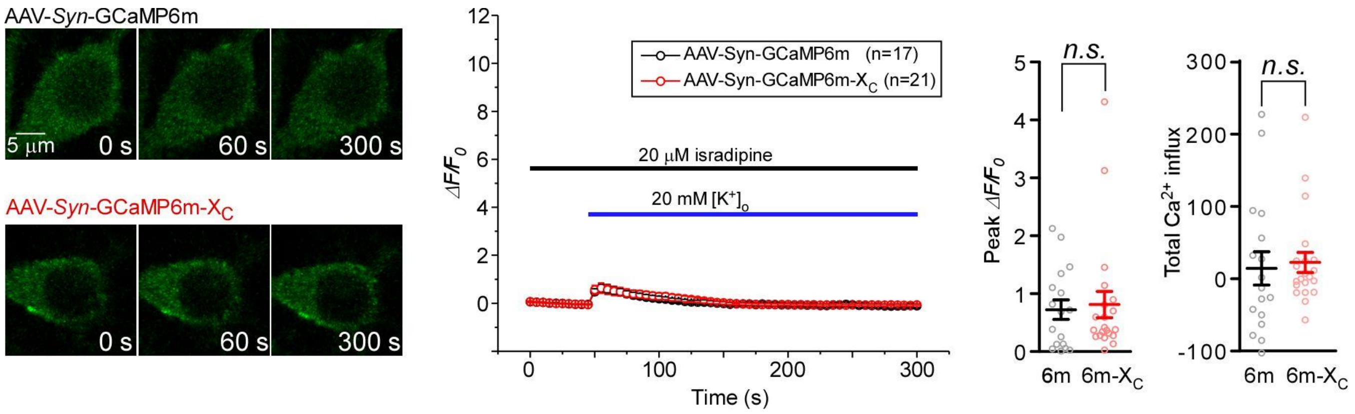

Supplementary Fig. 7 Ca^{2+} influx in response to 20 mM $[K^+]_o$ is dominated by Ca_V1.

Confocal fluorescent Ca^{2+} imaging for cortical neurons with AAV-*Syn*-GCaMP6m or AAV-*Syn*-GCaMP6m-X_C. Cortical neurons were treated with 20 μM of Ca_V1 specific blocker of isradipine for whole process of 20 mM $[K^+]_o$ stimulation. Most of calcium entry for both groups was reduced to similar low level by isradipine, evidenced by statistic summary of response curves, peak $\Delta F/F_o$ and total influx.

Standard error of the mean (S.E.M.) and Student's *t*-test (two-tailed unpaired with criteria of significance: *, $p<0.05$; **, $p<0.01$ and ***, $p<0.001$) were calculated when applicable, and *n.s.* denotes "not significant".

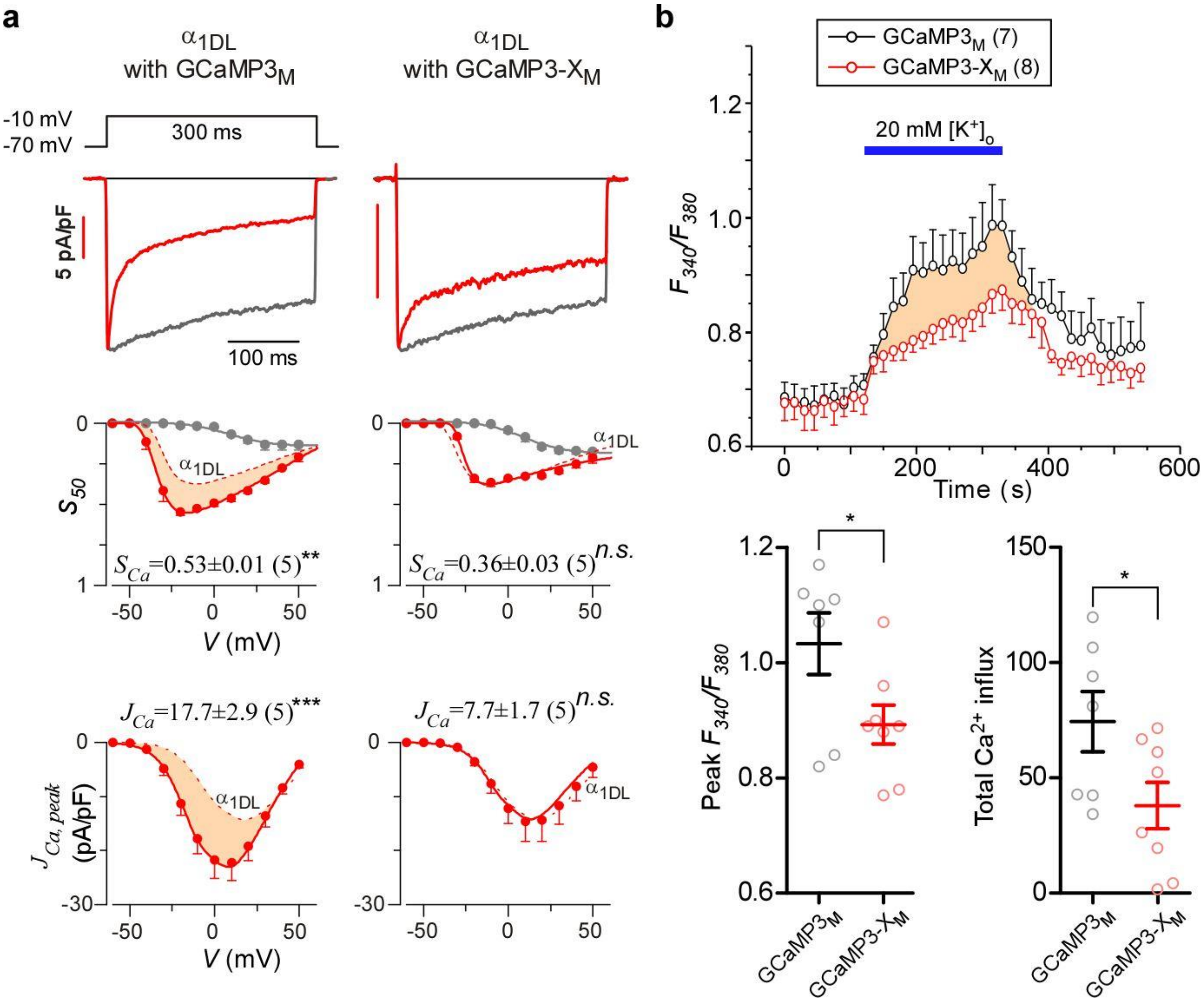

Supplementary Fig. 8 Validation of membrane-targeted version GCaMP3-X_M.

(**a**) Validation of GCaMP3-X_M with electrophysiological recordings of recombinant Ca_V1.3 channels. GCaMP3$_M$ and GCaMP3-X_M, both containing membrane-targeted Lck tags, were examined for the effects on α_{1DL} gating in HEK293 cells. Indexed with S_{Ca} and J_{Ca}, dotted lines represent the profiles of α_{1DL} control. The enhancement of α_{1DL} gating by GCaMP3$_M$ was not observed with GCaMP3-X_M. (**b**) Validation of GCaMP3-X_M with cortical neurons under Fura-2 ratiometric Ca^{2+} imaging. GCaMP-X_M avoided the abnormal enhancement of Ca^{2+} dynamics observed with GCaMP3$_M$. F_{340}/F_{380} and its area beneath, indicative of dynamic Ca^{2+} concentration and total Ca^{2+} influx respectively, were both significantly larger for GCaMP3$_M$ than GCaMP3-X_M, in response to extracellular stimuli of 20 mM $[K^+]_o$. Standard error of the mean (S.E.M.) and Student's *t*-test (two-tailed unpaired with criteria of significance: *, p<0.05; **, p<0.01 and ***, p<0.001) were calculated when applicable, and *n.s.* denotes "not significant".

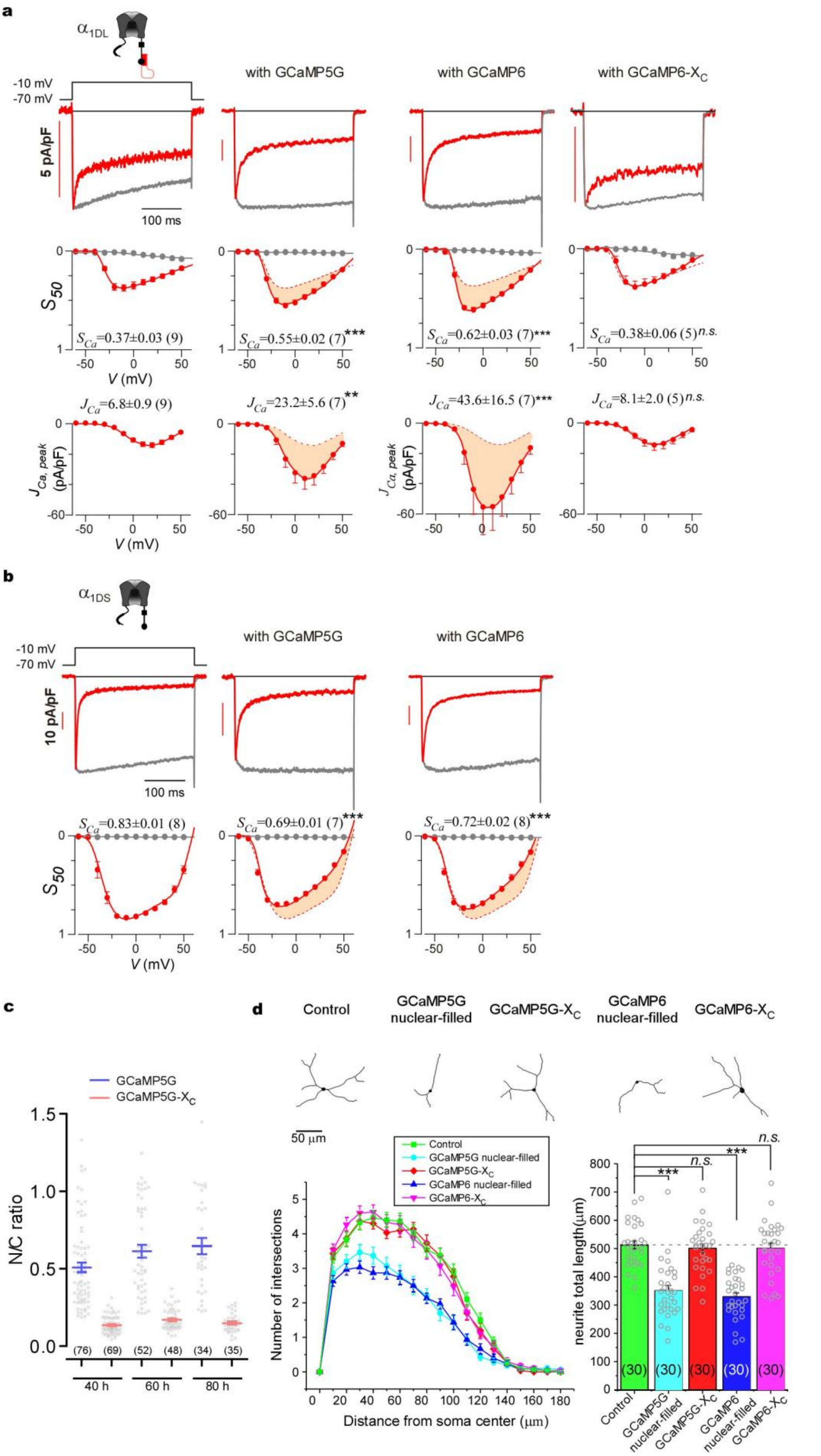

Supplementary Fig. 9 Effects of different versions of GCaMP on Ca_V1 gating and signaling.

(**a**, **b**) Effects on $Ca_V1.3$ gating. Similar to GCaMP3 (Fig. 2c, d), GCaMP5G and GCaMP6 (GCaMP6m) affected CDI (second row, S_{Ca}) and/or VGA (third row, J_{Ca}) of recombinant α_{1DL} (**a**) and α_{1DS} (**b**) to different extents (indicated by shaded areas in orange). In contrast, GCaMP6-X_C (GCaMP6m-X_C) reverted the above effects on α_{1DL}, consistent with GCaMP3-X_C (Fig. 4b). (**c**) Average N/C ratio of cortical neurons expressing GCaMP5G or GCaMP5G-X_C at different time points. GCaMP5G-X_C maintained at lower N/C ratio throughout, whereas GCaMP5G exhibited a time-dependent trend of nuclear invasion. (**d**) Nuclear-filled GCaMP5G and GCaMP6m exhibited significant neurite damages which were effectively avoided with GCaMP5G-X_C and GCaMP6m-X_C respectively. Representative tracing images (upper), Sholl analysis (lower left) and total neurite length per neuron (lower right) are shown and compared for the four different groups of neurons: YFP control, nuclear-filled GCaMP5G, GCaMP5G-X_C, nuclear-filled GCaMP6m and GCaMP6m-X_C.

Standard error of the mean (S.E.M.) and Student's *t*-test (two-tailed unpaired with criteria of significance: *, $p<0.05$; **, $p<0.01$ and ***, $p<0.001$) were calculated when applicable, and *n.s.* denotes "not significant".

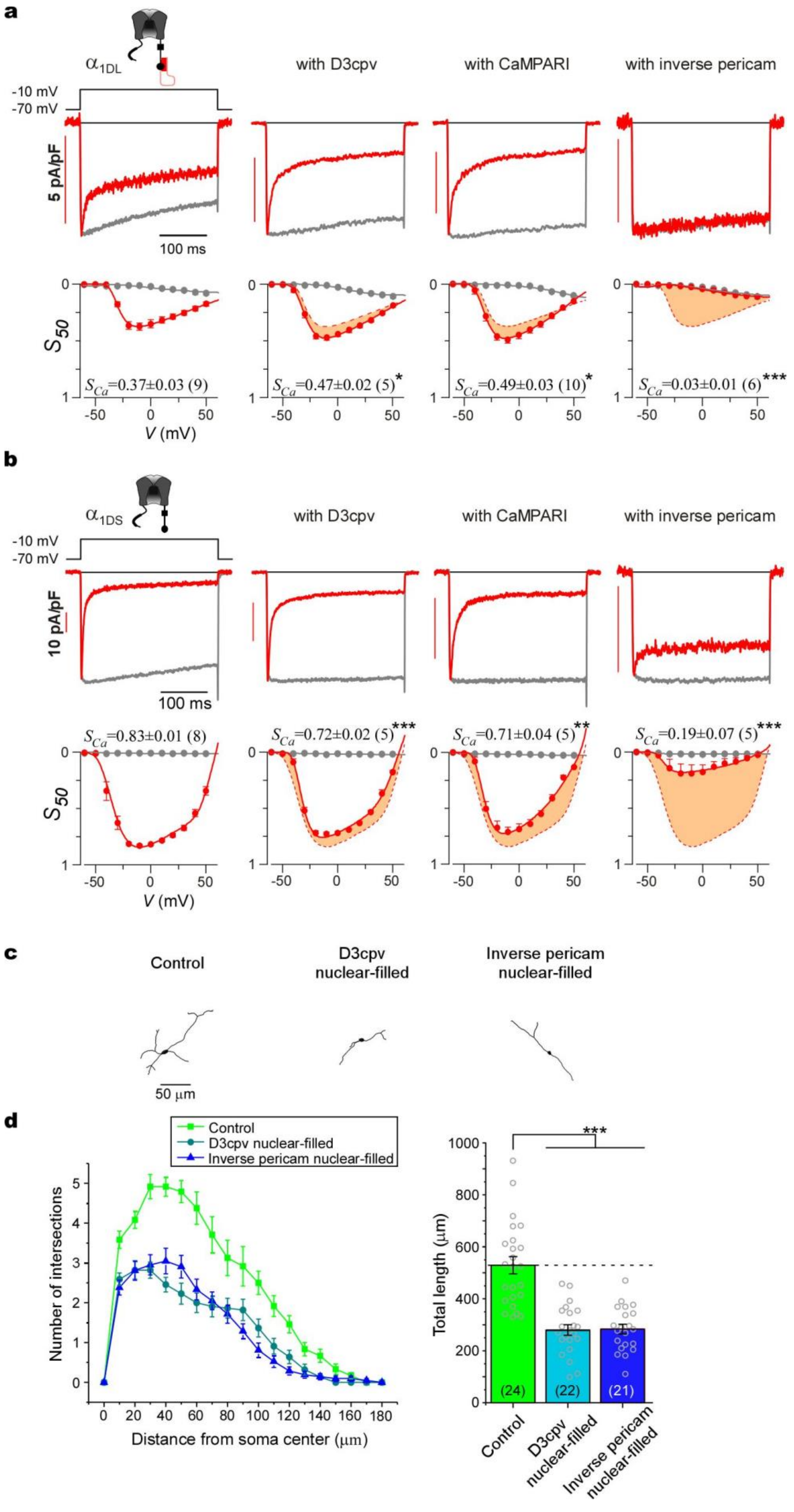

Supplementary Fig. 10 Similar side-effects by other CaM-based GECIs.

(**a**, **b**) Representative recording exemplars in HEK293 cells expressing short or long variant of $Ca_V1.3$ channels alone, or co-expression with various CaM-based GECIs, including D3cpv, CaMPARI, and inverse pericam. D3cpv, CaMPARI, and inverse pericam perturbed CDI of α_{1DS} and α_{1DL}. CaM enclosed within these GECIs could compete with endogenous (apo)CaM or DCT respectively, and also exhibited impaired Ca^{2+}/CaM signaling. Inverse pericam caused the most pronounced effects on Ca^{2+}/CaM, nearly knocking out CDI. CaMPARI containing a NES tag still exhibited clear effects on CDI. (**c**) Representative tracing images of cortical neurons with YFP and nuclear-filled D3cpv or inverse pericam. (**d**) Neurite growth quantified by Sholl analysis (left) and total neurite length (right). D3cpv and inverse pericam significantly damaged neurite outgrowth when present in nuclei.
Standard error of the mean (S.E.M.) and Student's *t*-test (two-tailed unpaired with criteria of significance: *, $p<0.05$; **, $p<0.01$ and ***, $p<0.001$) were calculated when applicable.

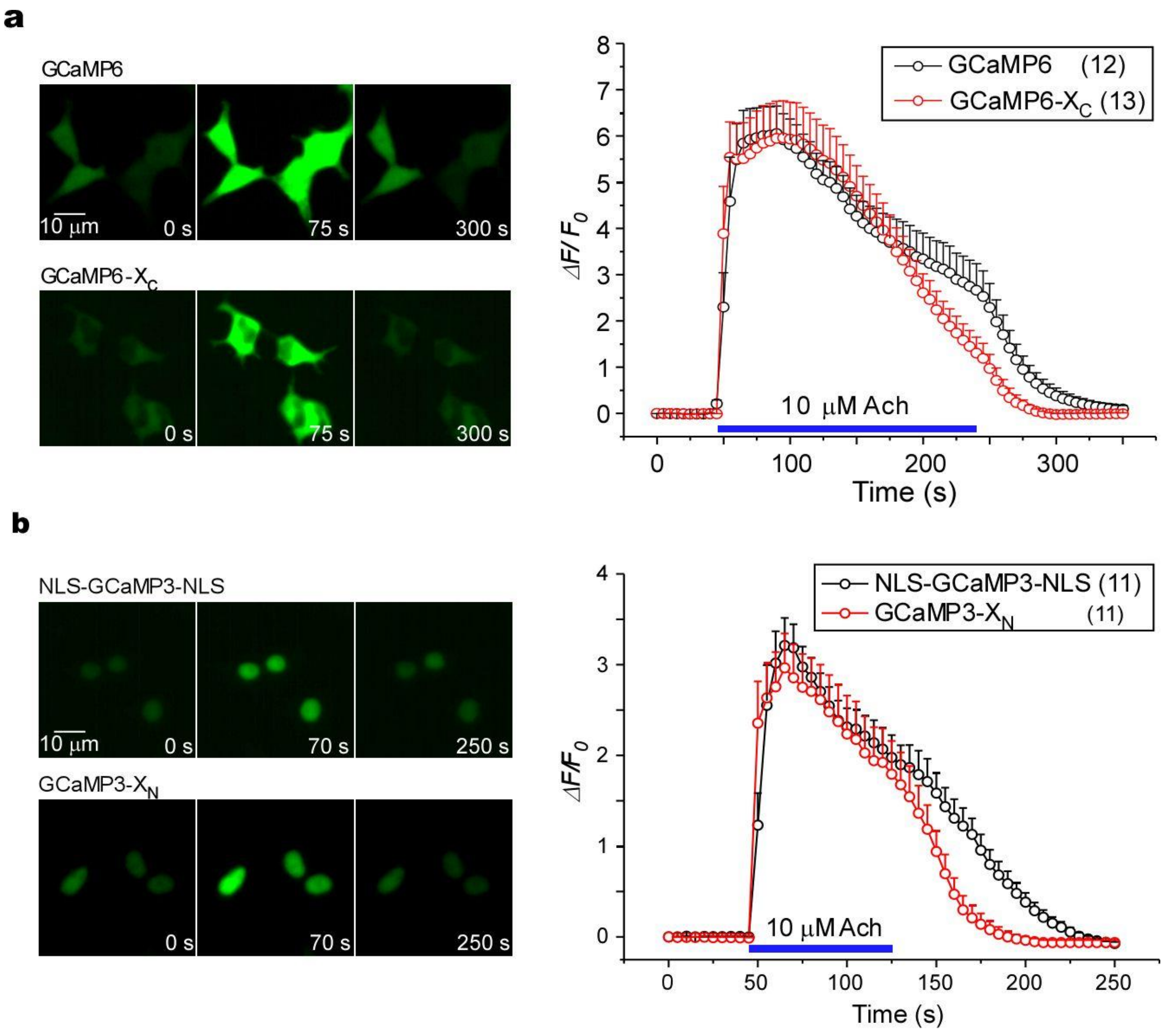

Supplementary Fig. 11 Performance of GCaMP *vs* GCaMP-X in monitoring Ca^{2+} dynamics of HEK293 cells.

(**a**) Exemplar images (left) at different phases: before (resting), during (peak) and after (washout) extracellular stimuli of 10 μM Acetylcholine (Ach). Averaged response curves across multiple cells (right) indicate that similar measurements (amplitude and kinetics) of cytosolic Ca^{2+} were obtained from GCaMP3-X_C and GCaMP3. (**b**) In response to 10 μM Ach, fluorescence signals acquired by GCaMP3-X_N and NLS-GCaMP3-NLS are closely resembled to each other, similarly depicting nuclear Ca^{2+} dynamics.

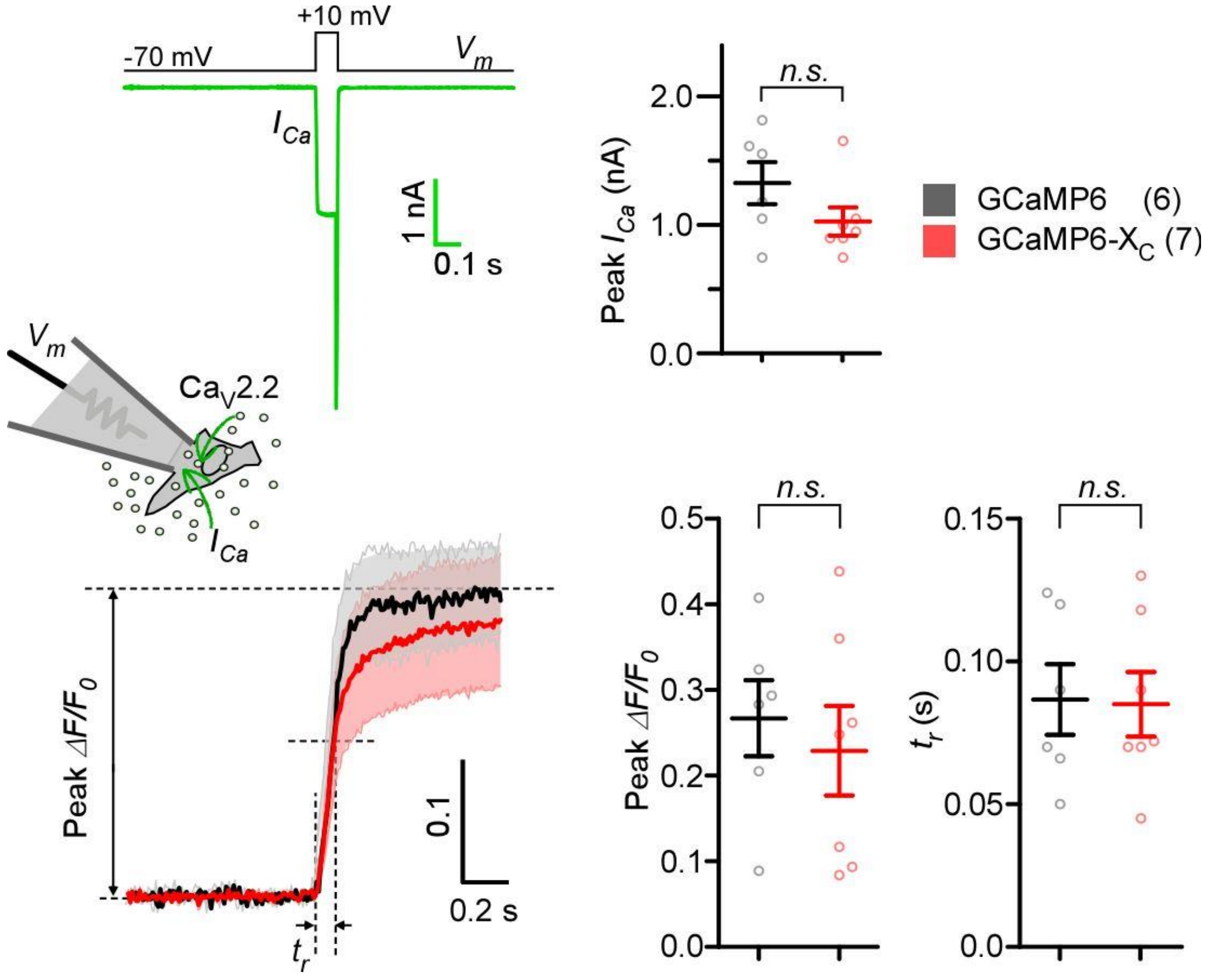

Supplementary Fig. 12 Fast onset kinetics of $Ca_V2.2$ influx measured by GCaMP and GCaMP-X.

HEK293 cells were co-transfected with $Ca_V2.2$ and GCaMP6m or GCaMP6m-X_C as the measurement platform to examine the performance of GCaMP and GCaMP-X sensors. Fast Ca^{2+} influx was induced by 100 ms of +10 mV depolarization that activated $Ca_V2.2$ (0.5 mM EGTA in recording pipette). GCaMP6m and GCaMP6m-X_C similarly depicted rapid and strong signals of Ca^{2+} fluorescence, with comparable values of peak $\Delta F/F_0$ and rise time (t_r, time to reach half of the peak).

Standard error of the mean (S.E.M.) and Student's *t*-test (two-tailed unpaired with criteria of significance: *, $p<0.05$; **, $p<0.01$ and ***, $p<0.001$) were calculated when applicable.

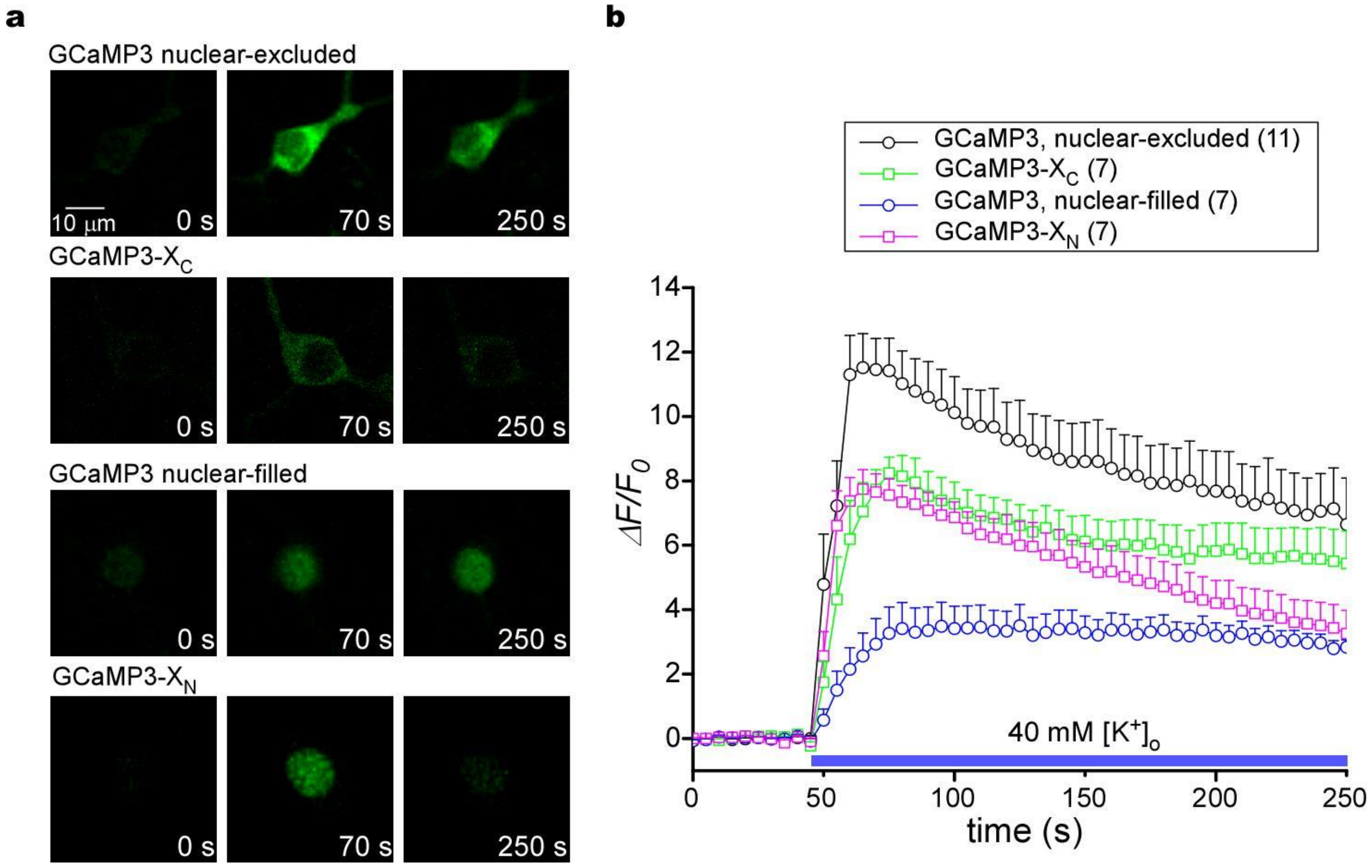

Supplementary Fig. 13 Confocal Ca^{2+} imaging in neurons to compare GCaMP3 with GCaMP3-X_C or GCaMP-X_N.

(**a**) Representative images at different phases: before (resting), during (peak) and end (later phases) of extracellular stimuli of 40 mM $[K^+]_o$. (**b**) Averaged curves indicative of Ca^{2+}-dependent fluorescence signals were compared among nuclear-excluded GCaMP3, GCaMP3-X_C, nuclear-filled GCaMP3 and GCaMP3-X_N.
